# Preventing yellow fever epidemics in Asian megacities: how can cities control mosquito-transmitted diseases?

**DOI:** 10.1080/23748834.2021.1899486

**Published:** 2021-03-29

**Authors:** Fiona C. Shenton, Steve W. Lindsay

**Affiliations:** Department of Biosciences, Durham University, Durham, UK

**Keywords:** Yellow fever, mosquitoes, cities

## Abstract

The COVID-19 pandemic has reminded us of the ever present threat from infectious diseases, this includes the ones we know about already and future unknowns. The mosquito-transmitted disease yellow fever has claimed thousands of lives over the centuries and it hasn’t gone away. It is still endemic in tropical areas of Africa and Latin America, where it is kept at bay through constant surveillance, mass vaccination campaigns and some natural immunity within local populations. Despite this there are serious outbreaks from time to time. The *Aedes* mosquitoes capable of transmitting the virus from person to person, are now widespread in warmer countries worldwide, moreover they thrive in urban areas. With increased international movement, the fear is that infected travellers could unwittingly introduce the virus into countries where people have little or no immunity. Densely populated Asian megacities are a major concern. There are simple measures citizens can take to protect themselves and their homes from the bite of infected mosquitoes, but city leaders must be at the forefront of a coordinated response bringing together diverse stakeholders to ensure a robust and sustainable defence.

Large-scale outbreaks of new infectious diseases have occurred throughout human history, often with devastating consequences (McMichael 2001). What has changed is that they are occurring more frequently than before (Woolhouse *et al*. 2008). And, as shown by the world’s response to the COVID-19 pandemic, we are poorly prepared for large-scale disease outbreaks (Horton 2020). Here we describe the potential threat posed by the mosquito-transmitted disease yellow fever. Yellow fever is not new, but there is a danger of it spreading into new areas, especially Asian cities. City leaders have an important part to play in mitigating against this peril.

Even within living memory (just) the present pandemic of COVID-19 is not unprecedented. In 1918 ‘Spanish’ flu caused the deaths of 17–50 million people (some put estimates even higher), far outstripping the numbers of young men killed in combat during World War 1 (Spinney 2017). More recently, the world has experienced epidemics of Human Immunodeficiency Virus/Acquired Immune Deficiency Syndrome (HIV/AIDS) in 1981, Severe Acute Respiratory Syndrome (SARS) in 2002–2004, Swine flu (H1N1) 2009–2010, Middle East Respiratory Syndrome (MERS) in 2012–2013 and Ebola from 2013–2016.

Of particular concern are those diseases transmitted by a common black and white urban mosquito, *Aedes aegypti* (Figure 1), probably found in every town and city throughout the tropics and sub-tropics. *Aedes*-transmitted diseases include dengue, chikungunya, Zika and yellow fever. Dengue, with the exception of COVID-19, is now the fastest growing infectious disease in the world. The number of global dengue cases has increased over eight fold within the last two decades, rising from 505,403 in 2000 to 4.2 million in 2019 (See https://www.who.int/news-room/fact-sheets/detail/dengue-and-severe-dengue, accessed 23/02/2021), demonstrating the capacity of this mosquito to transmit viruses.

**Figure 1.**
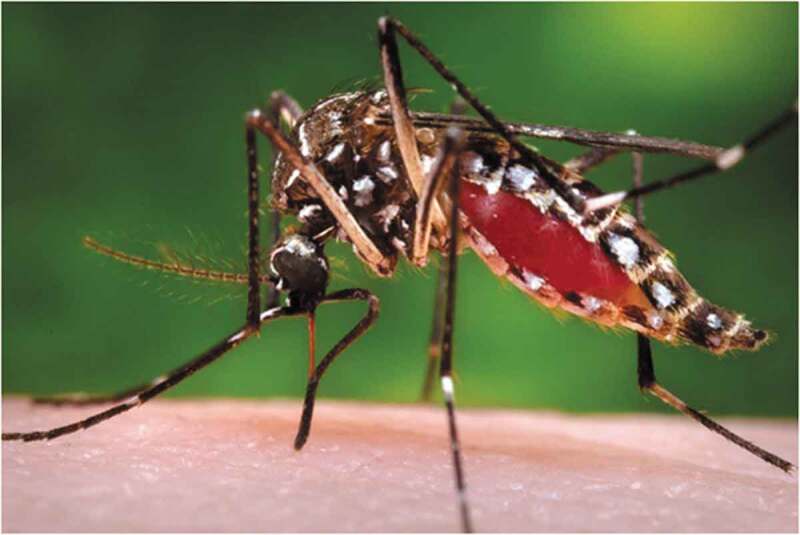
*Aedes aegypti*, formerly known as the yellow fever mosquito. Centers for disease control and prevention public health image library https://phil.cdc.gov/details_linked.aspx?pid = 9261.

Yellow fever is the most deadly of the *Aed*es-transmitted diseases and, is presently found in tropical Africa and Latin America. The disease derives its name from the jaundice which develops in severe cases (See https://www.who.int/news-room/fact-sheets/detail/yellow-fever, accessed 23/02/21). Symptoms appear rapidly, within three to six days, and include fever, muscle pain with prominent backache, headache, loss of appetite, and nausea or vomiting (Figure 2). Usually these symptoms disappear after a further three to four days, but a number of people enter a more dangerous phase of the disease. Within 24 hours of appearing to recover from the initial symptoms, the high fever returns and multiple body systems are affected, commonly the liver and kidneys. It is during this phase that patients develop jaundice and turn yellow. The liver damage also leads to dark urine, abdominal pain and vomiting. Bleeding can occur from the mouth, nose, eyes or stomach. Half of those who enter this second, distressing stage will die within 7–10 days.

**Figure 2.**
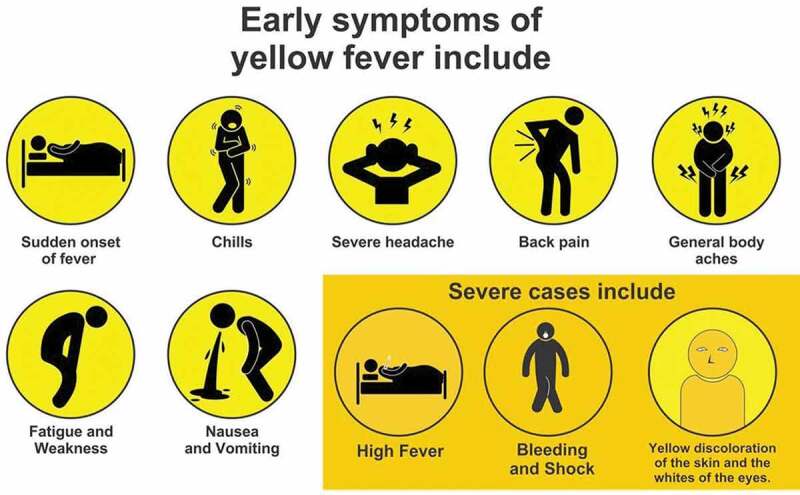
Symptoms of yellow fever.

https://www.mxpharmacy.co.uk/uploads/4/1/5/5/41,550,283/yellow-fever-symptoms_orig.jpg

Since many people are asymptomatic, the true case fatality rate (CFR) is difficult to determine, however, for those who become sick, 20–60% die (Monath and Vasconelos 2015). At the end of the 19th century, when the United States invaded Cuba, for every soldier who died in battle, 13 died of yellow fever (Bryan *et al*. 2004). This compares with the current estimate for the CFR from SARS-CoV-2 of about 1.4% in China (Verity *et al*. 2020).

Many of us are now familiar with the concept of the reproduction number (R_0_), the average number of secondary infections produced by a single infected person. Estimates of R_0_ in the case of mosquito-transmitted diseases are trickier to estimate. An infectious mosquito may give rise to any number of infectious humans depending on the feeding rate, transmission efficiency, and how long the mosquito lives for. Nonetheless, for a pathogen, hitching a ride in an insect or tick can allow it to locate a host more easily and can therefore increase R_0_ by orders of magnitude compared with many directly transmitted diseases (Rogers and Packer 1993), like measles, mumps and COVID-19. A recent study suggested R_0_ could rise as high as 90 for yellow fever (Johansson *et al*. 2012). While the study authors thought that the R_0_ in most yellow fever epidemics would likely to be much lower, closer to 1; the potential for such high transmission is truly alarming, particularly in populations that are unvaccinated. Despite there being an extremely effective vaccine for yellow fever, large epidemics occur in heavily populated areas where people have little or no immunity, due to lack of vaccination.

The yellow fever mosquito, *Ae. aegypti*, was originally a tree-hole breeding mosquito found in the forests of West Africa. But, it has adapted to an urban existence by laying its eggs in small bodies of water such as collect in discarded containers, water containers, underground culverts, guttering or old tyres. Towns and cities offer perfect habitats for this mosquito with a plethora of breeding sites and ready access to a human bloodmeal. In Asia, *Ae. aegypti* is now well-entrenched (Kraemer *et al*. 2019) and extremely capable of transmitting viruses, as shown by the rapidly increasing number of dengue cases in the region. Indeed dengue fever may be viewed as a useful proxy for identifying populations where yellow fever could be transmitted. Worryingly, we know from laboratory studies that Asian strains of *Ae. aegypti* can transmit yellow fever (Souza-Neto *et al*. 2019). Should that happen outside the laboratory, in densely populated Asian towns and cities where *Aedes* mosquitoes are abundant, the consequences would be catastrophic. The map in Figure 3 shows cities with populations over one million overlaid on the worldwide distribution of *Ae. aegypti*. Megacities clustering in India and China are of particular concern because populations here are neither naturally immune (having not been previously exposed to the virus) nor vaccinated. The drivers are 1. Presence of urban, human-biting mosquitoes, especially *Aedes aegypti* 2. Large non-immune, densely packed populations 3. Increased travel between countries where yellow fever is endemic and those large, non-immune urban populations. How can we protect Asian towns and cities from yellow fever? How do we do this in a megacity?

**Figure 3.**
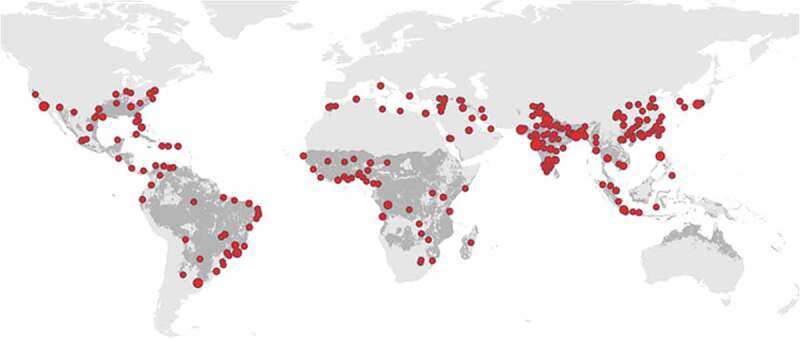
Cities and the distribution of the yellow fever mosquito. Predicted distribution of *Aedes aegypti* in 2015 (grey) and cities of over one million inhabitants (red). (Reproduced by kind permission of Dr Nick Golding, Curtin University, Perth, Australia).

The drivers of yellow fever epidemics are urbanization and rapid movement of people over great distances contributing to large urban outbreaks, notably in Angola in 2015 − 16 when the World Health Organisation (WHO) declared a global threat (See https://www.who.int/csr/don/22 March 2016-yellow-fever-angola/en/, accessed 23/02/2021). There was real fear that the disease might spill over into China. There were indeed confirmed reports of Chinese workers infected with yellow fever in Angola, returning home to China. It was only a matter of great good fortune, that the disease did not spread from these individuals into the wider population, potentially 1.8 billion mostly unvaccinated people in Asia (Wilder-Smith and Monath 2017). Projections show that another 2.5 billion people will be added to urban areas by 2050 (United Nations report, 2018 See https://www.un.org/development/desa/en/news/population/2018-world-urbanization-prospects.html, accessed 23/02/2021), with approximately 90% of this increase occurring in Asia and Africa. Most of these countries do not vaccinate those at risk. Whilst WHO has an emergency stockpile of 18 million doses of yellow fever vaccine, world supplies are under pressure. In 2015 − 2016, Angola was grappling with the worst yellow fever outbreak in decades (WHO.int). With the disease spreading to neighbouring countries, the death toll rising, and a shortage of vaccine, WHO agreed to the vaccine being used at a fractional dose as an interim measure in order to make supplies go further. This was a desperate state of affairs. In response to this threat, WHO (in partnership with UNICEF and Gavi) developed the Eliminate Yellow Fever Epidemics (EYE) Strategy 2017–2026. Worryingly, EYE Strategy implementation has been severely impacted by COVID-19, with all 2020 campaigns postponed by several months (WHO Weekly epidemiological record No 34, 2020). Control of a recent (December 2020) outbreak of yellow fever in Nigeria has been hampered by the current focus on the COVID-19 pandemic response, and the relative proximity of the states affected to Lagos, an urban environment with a large unvaccinated population, is an added concern (See https://www.afro.who.int/news/responding-yellow-fever-outbreak-nigeria-amidst-global-pandemic, accessed 23/02/2021)

Alongside measures more traditionally seen as ‘health’ interventions such as vaccination, the EYE Strategy emphasises the importance of building resilient urban centres. House improvements and good environmental management are integral to reducing disease transmission (Figure 4).

**Figure 4.**
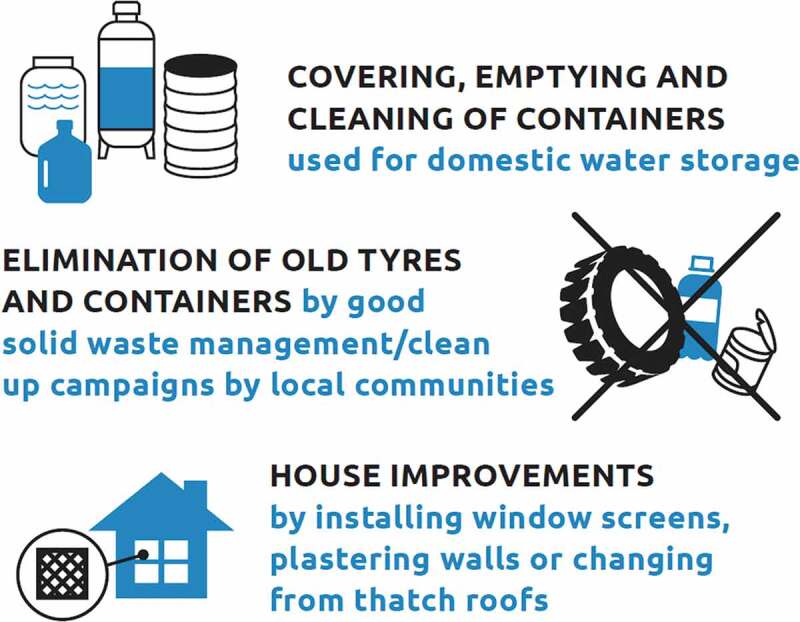
WHO recommendations for the control of the yellow fever mosquito.

Adapted from Global vector control response 2017–2030: A strategic approach to tackle vector-borne diseases WHO/HTM/GVCR/2017.01 https://apps.who.int/iris/bitstream/handle/10,665/259,002/WHO-HTM-GVCR-2017.01-eng.pdf;jsessionid = 0678B3844DED1434E47261C7745312CB?sequence = 1

Keeping homes and their surroundings clear of rubbish reduces potential mosquito aquatic habitats. Underground water sources such as culverts, covered drains and soakaways, should be designed so as not to become mosquito breeding sites. Similarly a safe, reliable water supply without leaks and the need to store water in temporary containers; reduces the risk. Inside the house, simple improvements, including blocking of eaves, well-fitted doors and windows, and mosquito screening; can reduce mosquito entry and prevent indoor biting. At the same time it is important that houses remain well ventilated and cool so that inhabitants are comfortable indoors and will sleep under insecticide treated bednets where necessary. Based on the best evidence to date, we have recently drawn up recommendations, for simple steps to protect people from mosquitoes in and around their homes (Lindsay *et al*. 2021) (Figure 5).

**Figure 5.**
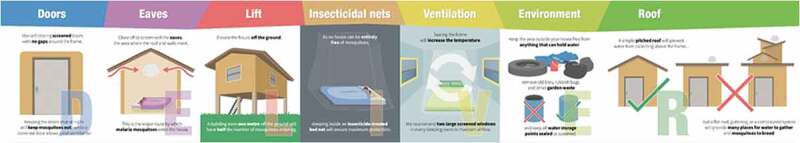
The DELIVER mnemonic: our recommendations for protecting people from mosquitoes in and around their homes. https://doi.org/10.1098/rstb.2019.0814

While some of these measures (such as screening of windows and doors, and covering water containers) can be implemented by householders and communities themselves, it falls to city leaders to coordinate responses across communities and the wider population. Essentially mosquito control is an environmental issue, requiring a multi-sectoral response. Box 1 lists **Essential reading** for those seeking in depth guidance on recommended interventions.

**Box 1.** Essential reading.

1. Lindsay *et al*. (2021) Recommendations for building out mosquito-transmitted diseases in sub-Saharan Africa: the DELIVER mnemonic. Philosophical Transactions B 376(1818):20,190,814. DOI: https://doi.org/10.1098/rstb.2019.0814
*A paper with specific recommendations for house and environmental improvements to protect people from mosquito bites.*
2. World Health Organisation. Global Vector Control Response 2017–2030. Geneva: WHO; (2017), ISBN 978–92-4-151,297-8.
*A really important document from the World Health Organisation describing in detail how to control disease vectors including mosquitoes.*
3. Sim *et al*. (2020) A greener vision for vector control: The example of the Singapore dengue control programme. PLOS Neglected Tropical Diseases, 14(8), e0008428 DOI: https://doi.org/10.1371/journal.pntd.0008428
*Singapore as a unique exemplar of how a city coordintaes effective mosquito control through a multisectoral approach.*

Community mobilisation to reduce sources of mosquitoes has been shown to be effective in a number of studies conducted in Central and South America, and Asia (Alvarado-Castro *et al*. 2017). These community- based interventions were all carried out alongside the local authorities usual mosquito/disease control programmes, so the benefits were additive. They are detailed and complex in their approach, first discussing the problem with stakeholders and involving them in the planning of interventions. Interventions commonly involve community-based environmental management approaches such as provision of water container covers through community actors, clean-up campaigns, and dissemination of dengue (or other mosquito-borne disease) information through schoolchildren or other groups. Importantly, they include working with providers of local services such as solid waste removal. The COVID-19 pandemic has highlighted the key role of city leaders in controlling disease outbreaks and maintaining essential services. Effective communication across sectors is required: health, mosquito control, housing, water & sanitation, and waste removal, and this must go alongside sound local knowledge of the situation on the ground.

Singapore’s impressive dengue control programme (Sim *et al*. 2020) is unique in bringing together all these elements, providing an exemplar of good practice. Although this programme predates WHO’s Global Vector Control Strategy (WHO, GVCR 2017 − 2030) it aligns closely with the GVCR approach. With Singapore’s National Environment Agency at its heart, the programme focusses on reduction of *Aedes* mosquito aquatic habitats through multi-sectoral engagement bringing in both government and nongovernment players along with communities (Figure 6). There is great attention to detail: households are regularly inspected and householders can be fined for having open water containers, or allowing water to pool around house plants, accumulated rubbish or other places. Roof gutters are banned and novel washing lines, constructed with holders which do not collect water, have been designed. Solid waste management is also an important element, ensuring removal of artificial containers which could become habitats for *Aedes* mosquitoes. Approximately 60% of Singapore’s waste is recycled and the remainder incinerated. The ash generated by incineration is added to an artificial offshore island, Semakau Island, contributing to land reclamation.

**Figure 6.**
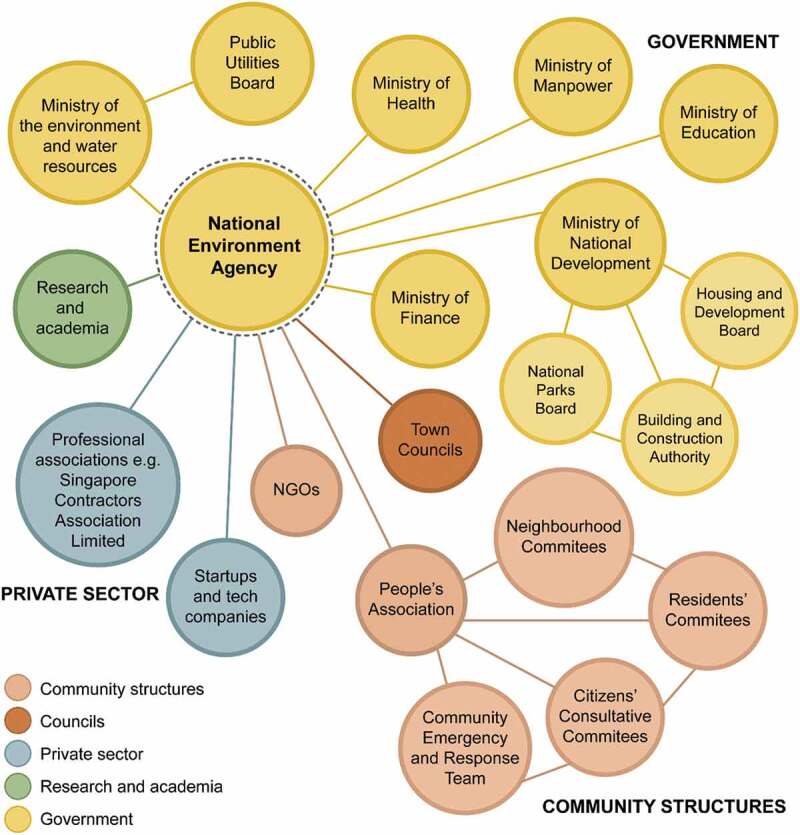
Collaboration between Singapore’s national environment agency and other sectoral stakeholders. NGO, nongovernmental organisation.

https://doi.org/10.1371/journal.pntd.0008428.g002

In addition constant surveillance is essential with clear lines of communication to report problems, alongside well planned and practiced responses to contain increases in mosquito populations as soon as they arise. In order for cities to be able to take on these responsibilities, power must be devolved from central government to city leaders alongside the fiscal resources to support them.

The United Nations’ Sustainable Development Goals (SDGs) also emphasise the necessity for cross-sectoral collaboration. The SDGs of most relevance to combatting mosquito-transmitted diseases such as yellow fever are Goal 3: Good Health and Wellbeing, Goal 11: Sustainable Cities and Communities and Goal 17: Partnerships to achieve the Goals. These SDGs are the drivers underpinning the United Nations organisations recommendations on health, housing and resilience. For practitioners from the built environment the UN-Habitat’s ‘New Urban Agenda’ (UN, 2017 Habitat III Quito 17–20 October 2016 https://habitat3.org/the-new-urban-agenda/) and the UN Office for Disaster Risk Making Cities Resilient Reduction’s ‘Making Cities Resilient 2030’ (See https://www.unisdr.org/campaign/resilientcities/, accessed 23/02/2021) offer important guidance.

## Summary

- Asia is home to cities with millions of people.
- The mosquito *Aedes aegypti* is abundant in cities and towns throughout the region. It is the world’s most efficient transmitter of arboviruses, including yellow fever which kills 20–60% of people who fall sick with the disease.
- Few people in Asia are vaccinated against yellow fever; while a very effective vaccine exists, supplies fall well short of what would be required to cover the total population potentially at risk.
- A yellow fever pandemic in an Asian city would claim 1,000s of lives.
- WHO recommends that mosquito populations should be controlled by combining good environmental management to reduce their aquatic habitats, with housing improvements to prevent mosquitoes entering dwelling places.
- Effective control relies on strong multi-sectoral collaboration with good communication between those in the health and built environment sectors.
- Power structures must be decentralised and authority devolved to city leaders alongside the necessary resources.

This approach has the added benefit of building resilience against all mosquito-transmitted diseases – including the new ones that we do not yet know about and for which we do not have treatments or vaccines. The **B**uilding **O**ut **V**ector-borne diseases in sub-Saharan **A**frica (BOVA) Network (www.bovanetwork.org) is currently working in partnership with other organisations to support a network of Commonwealth city leaders to address vector-borne (especially mosquito-transmitted) and Neglected Tropical Disease (NTD) prevention in cities. As devastating as a yellow fever pandemic would certainly prove to be, it might not even be the worst we could encounter. Prevention must be the priority, not vain attempts to close the stable door once the horse has bolted.
